# Phosphorylation of a serine/proline-rich motif in oxysterol binding protein-related protein 4L (ORP4L) regulates cholesterol and vimentin binding

**DOI:** 10.1371/journal.pone.0214768

**Published:** 2019-03-29

**Authors:** Antonietta Pietrangelo, Neale D. Ridgway

**Affiliations:** 1 Department of Biochemistry and Molecular Biology, Atlantic Research Center, Dalhousie University, Halifax, Nova Scotia, Canada; 2 Department of Pediatrics, Atlantic Research Center, Dalhousie University, Halifax, Nova Scotia, Canada; Simon Fraser University, CANADA

## Abstract

The family of oxysterol binding protein (OSBP) and OSBP-related proteins (ORPs) mediate sterol and phospholipid transfer and signaling at membrane contact sites (MCS). The activity of OSBP at MCS is regulated by phosphorylation, but whether this applies to ORPs is unknown. Here we report the functional characterization of a unique proline/serine-rich phosphorylation motif (S_762_SPSSPSS_769_) in the lipid binding OSBP-related domain of full-length ORP4L and a truncated variant ORP4S. Phosphorylation was confirmed by mass spectrometry and [^32^P]PO_4_ incorporation, and *in silico* and *in vitro* assays using purified ORP4L identified putative proline-directed kinases that phosphorylate the site. The functional significance of the phospho-site was assessed by mutating serine 762, S763, S766 and S768 to aspartate or alanine to produce phosphomimetic (S4D) and phosphorylation-deficient (S4A) mutants, respectively. Solution binding of 25-hydroxycholesterol and cholesterol by recombinant ORP4L-S4D and -S4A was similar to wild-type but ORP4L-S4D more effectively extracted cholesterol from liposomes. ORP4L homo-dimerization was unaffected by phosphorylation but gel filtration of ORP4L-S4D indicated that the native conformation was affected. Confocal microscopy revealed that ORP4L-S4D also strongly associated with bundled vimentin filaments, a feature shared with ORP4S which lacks the PH and dimerization domains. We conclude that phosphorylation of a unique serine/proline motif in the ORD induces a conformation change in ORP4L that enhances interaction with vimentin and cholesterol extraction from membranes.

## Introduction

Maintaining the heterogeneous composition of mammalian cellular membranes requires the transport of lipids from sites of synthesis to final destinations. Bulk transport of lipids between donor and acceptor membranes occurs by vesicular or fusion mechanisms whereas single lipid molecules are moved by lipid transport proteins (LTPs) (reviewed in [1]). Oxysterol binding protein (OSBP) and OSBP-related proteins (ORPs) constitute a 12-member family of mammalian LTPs of variable tissue expression, ligand specificity, subcellular localization and function (reviewed in [2]). The ORPs share a common C-terminal ligand binding OSBP-related domain (ORD), which binds cholesterol, oxysterols and phospholipids. Additional pleckstrin homology (PH), two phenylalanines in an acidic tract (FFAT) and ankyrin domains mediate interaction of OSBP/ORPs at contact sites between closely opposed membranes to facilitate lipid transfer and signalling activities. For example, ORP5 and ORP8 exchange phosphatidylserine and phosphatidylinositol 4-phosphate (PI(4)P) [3] or phosphatidylinositol 4,5-bisphosphate (PI4,5)P_2_ [4] at plasma membrane (PM)/endoplasmic reticulum (ER) contact sites, OSBP mediates PI(4)P and cholesterol exchange at ER/Golgi contacts [5], ORP2 transfers cholesterol and PI(4,5)P_2_ from late endosomes/lysosomes to the ER and PM [6] and ORP1 cholesterol transfer from the late endosomes lysosomes [7] is regulated by PI(4,5)P2 and phosphatidylinositol 3,4-bisphosphate [8].

ORP4 (OSBP2) is phylogenetically related to OSBP, binds cholesterol and PI(4)P and interacts with vesicle–associated membrane protein-associated protein (VAP) in the ER. Three promoter and/or splice variants are expressed from *OSBP2*; a full-length version with FFAT and PH domains (ORP4L), and two N-terminal truncated forms with partial (ORP4M) or complete (ORP4S) deletion of the PH domain. Unlike other ORPs, ORP4 has an essential role in the survival and proliferation of immortalized and transformed cells [9, 10]. shRNA silencing of all three variants in HeLa, HEK293 and non-transformed rat intestinal epithelial cells promoted growth arrest and apoptosis [10]. Similarly, a class of antineoplastic saponins called the ORPphilins are cytotoxic to an array of cancer cell lines, an activity that is mediated by binding the ORD of ORP4L and/or OSBP [11]. In T-cell acute lymphoblastic leukemia, PM-localized ORP4L supports increased cellular respiration and survival by scaffolding a PLCβ3/CD3ε/Gαq11 complex that produces inositol-triphosphate and triggers calcium release from ER stores [12]. Mechanistically, ORP4L binds PI(4,5)P_2_ at the PM and presents the substrate to PLCβ3 for hydrolysis [13]. ORP4L is also localized to the Golgi apparatus by a sterol/OSBP-dependent mechanism and is involved in the maintenance of organelle structure and PI(4)P content [14]. As well, ORP4S and ORP4M constitutively aggregate vimentin intermediate filaments, and the ORP4 ORD binds vimentin *in vitro* [15]. ORP4L with a PH domain mutation that abolished PI(4)P binding also aggregated vimentin [10], suggesting that a functional PH domain negatively regulates the ORP4/vimentin interaction.

**Fig 1:**
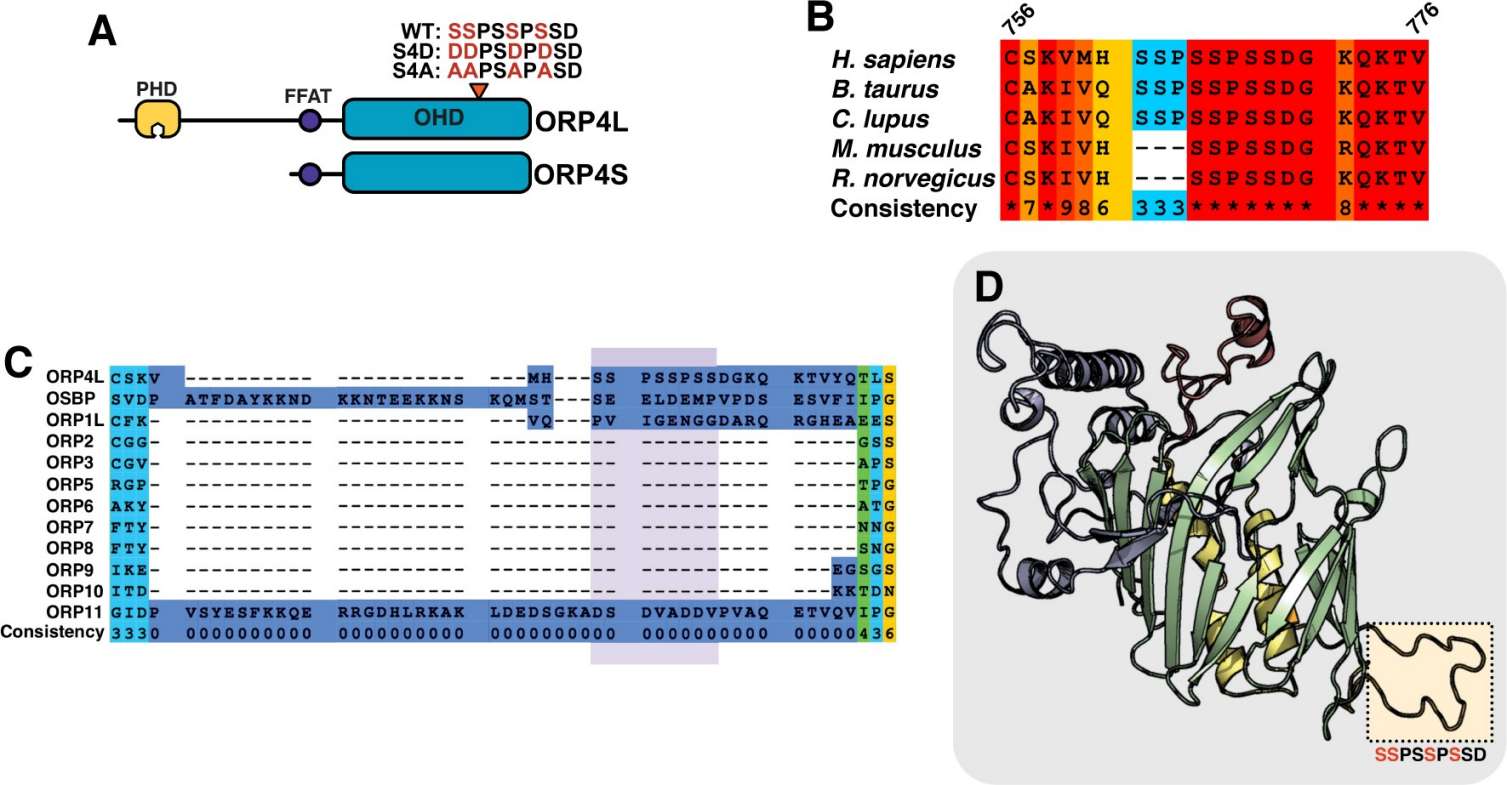
Identification of a serine/proline-rich phosphorylation motif in ORP4. (**A**) Domain organization of ORP4L and ORP4S showing the position of the serine/proline-rich motif. (**B**) PRALINE sequence alignment (http://www.ibi.vu.nl/programs/pralinewww/) [21] of the serine/proline phosphorylation site in ORP4 from *H*. *sapiens*, *R*. *norvegicus*, *M*. *musculus*, *B*. *taurus*, and *C*. *lupus*. The consistency (conservation) at each residue is scored from 1–10. (**C**) PRALINE sequence alignment of the phosphorylation site in human OSBP and ORPs (highlighted in purple). (**D**) SWISS-MODEL homology model of the ORP4L ORD based on the crystal structure for Osh3p (PDB: 4INQ). The ORD is an incomplete β-barrel (green) flanked by two central helices (yellow), an α-helical lid (red) and N-terminal region (blue). The predicted loop containing the phosphorylated serine residues is highlighted.

Curated phospho-proteome databases indicate that OSBP/ORPs are extensively phosphorylated, indicating a potential role in lipid transfer and signaling functions at membrane contact sites. For example, OSBP phosphorylation on a protein kinase D site [16], and on two serine-rich motifs adjacent to the FFAT and PH domains [17], influences ER-Golgi association, sterol binding and VAP interaction. Despite its close phylogenetic relationship to OSBP, ORP4L lacks these phosphorylation sites but harbours a unique proline/serine-rich phosphorylation motif in the ORD that could affect activity (Fig 1A). We verified phosphorylation of the site and used site-specific mutants of ORP4L to show that phosphorylation enhances cholesterol extraction and vimentin interaction by ORP4L such that it mimics the activity of the ORP4S variant.

## Materials and methods

### Materials

Cholesterol and 25-hydroxycholesterol were purchased from Steraloids (Newport, RI). [1,2-^3^H]Cholesterol, 25-[26,27-^3^H]hydroxycholesterol and [^32^P]PO_4_ were purchased from PerkinElmer Life Sciences (Boston, MA). [^14^C]-Dipalmitoyl-phosphatidylcholine was purchased from American Radiolabeled Chemicals (St. Louis, MO). Phospholipids were purchased from Avanti Polar Lipids (Alabaster, AL). Antibodies against giantin and polypeptide N-acetylgalactosaminyltransferase (GALNT) (BioLegend, San Diego CA), vimentin (Abcam, Cambridge UK) and V5 (BioRad, Raleigh NC) were used for immunofluorescence microscopy and immunoblotting. Alexa Fluor 488- and Alexa Fluor 594-conjugated secondary antibodies (Molecular Probes, Eugene OR) and IRDye 680LT- and IRDye 800CW-conjugated secondary antibodies (LI-COR Biosciences, Lincoln NE) were used for immunofluorescence and immunoblotting, respectively.

### Cell culture and transfections

HeLa, U2OS and HepG2 cells were cultured in DMEM supplemented with 10% (v/v) FBS. Cells were transiently transfected with plasmids using Lipofectamine 2000 (Invitrogen, Carlsbad CA) for 48 h. Sf21 cells were cultured in suspension at 27°C with constant shaking (150 rpm) in SF900-II medium supplemented with 5% (v/v) FBS, G418 (10 μg/mL), fungizone (0.25 μg/mL) and penicillin-streptomycin-glutamine (100 U/mL).

### Site directed mutagenesis and plasmid construction

Site-directed mutagenesis of pcDNA-ORP4L-V5-His was used to convert serine 762, 763, 766 and 768 to aspartate (S4D) or alanine residues to create phosphomimetic (ORP4L-S4D) and phosphorylation-resistant (ORP4L-S4A) mutants, respectively. ORP4S-S4D-V5 and ORP4S-S4A-V5 constructs were made by endonuclease digestion of pcDNA-ORP4L-V5-S4D and -S4A with AfeI and EcoRV and ligation of the fragment into pcDNA3.1-V5-His digested with EcoRV. A similar strategy was used to introduce S4A and S4D mutations into pEGFP-ORP4L. To create baculoviral constructs, S4A and S4D mutations were made by site-directed mutagenesis of pENTR/D-TOPO-ORP4L [10] and inserted into linear baculovirus DNA by recombination using the Gateway cloning system to introduce a C-terminal 6xHis-tag (Invitrogen).

### Liquid chromatography-tandem mass spectrometry analysis of phosphorylation

HeLa cells transiently expressing ORP4L-V5 were incubated with 0.5 μM okadaic acid for 2 h prior to lysis in RIPA buffer (10 mM sodium phosphate buffer, 150 mM NaCl, 2 mM EDTA, 2 mM EGTA, 10 mM NaF, 1 mM sodium pyrophosphate, 0.3% Triton X-100, pH 7.4). Lysates were immunoprecipitated with a V5 monoclonal antibody and protein A-Sepharose, resolved by SDS-8%PAGE and visualized with GelCode Blue. The band corresponding to ORP4L-V5 was excised and digested using an automatic digestion robot (ProGest, Genomic Solutions) [18]. LC-MS/MS was performed using a nanoflow liquid chromatography system (Ultimate3000, ThermoScientific) interfaced to a hybrid ion trap-orbitrap high resolution tandem mass spectrometer (VelosPro, ThermoScientific) operated in data dependent acquisition mode. Briefly, 1 μL of each sample was injected onto a capillary column (C18 Onyx Monolithic, 0.10 x 150 mm, Phenomenex) at a flow rate of 300 nL/min. Samples were electro-sprayed at 1.2 kV using a dynamic nanospray probe with fused silica non-coated emitters (20 μm ID with 10 μm ID tip, PicoTip Emitter from New Objective). Chromatographic separation was carried out using 60 min linear gradients (mobile phase A: 0.1% formic acid in water, mobile phase B: 0.1% formic acid in acetonitrile) from 3% B to 35% B over 40 minutes, then increasing to 95% B over 5 minutes. MS/MS spectra were acquired using both collision-induced dissociation (CID) and higher-energy collisional dissociation (HCD) for the top 10 peaks in the survey 30000-resolution MS scan. The *raw* files were acquired (Xcalibur, ThermoFisher) and exported to Proteome Discoverer 2.0 (ThermoFisher) software for peptide and protein identification using the SequestHT DB search engine (full trypsin digestion with 2 maximum missed cleavages, 10 ppm precursor mass tolerance and 0.8 Da fragment mass tolerance). Database searching was done using the UniprotKB human database. Oxidized methionine residues and phosphorylation of serine, threonine and tyrosine were selected as dynamic (variable) modifications; carbamidomethyl cysteine was selected as fixed modification.

### Recombinant ORP4L expression and purification

SF21 cells in suspension culture were transduced with baculovirus (MOI of 0.1) for 72 h, collected by centrifugation and stored at -80°C. Cell pellets were resuspended in wash buffer (10 mM Tris, 150 mM NaCl, 30 mM imidazole, pH 7.4) supplemented with EDTA-free protease inhibitor cocktail (Roche) and dispersed by passage through 18 and 25 G needles. His-tagged ORP4L was purified from lysates using metal affinity chromatography and concentrated by centrifugation in a Millipore Amicon column (30 K cut-off), diluted in storage buffer (10 mM Tris, 150 mM NaCl) and stored at -80°C [10].

### Sterol binding liposome extraction assays

Binding of recombinant ORP4L, ORP4L-S4A or ORP4L-S4D (8 pmol) with [^3^H]25OH was performed in the presence or absence of 40-fold excess unlabelled sterol to determine specific binding as previously described [17]. Binding constants were derived from Scatchard plots (GraphPad Prism v7).

For PI(4)P extraction assays, radiolabelled PI(4)P was prepared by incubating HeLa cells with [^32^P]PO_4_ (0.5 mCi/ml) for 16–20 h in phosphate-free DMEM supplemented with 2% FCS as previously described [17]. Liposomes for PI(4)P extraction contained phosphatidylcholine (PC)/phosphatidylethanolamine (PE)/phosphatidylserine (PS)/lactosyl-PE/[^32^P]PI(4)P (60:20:10:10:2000 DPM/assay), and were prepared by extrusion through 400 nM pore membranes using the AVESTIN LiposoFast system (Ottawa, ON). Liposomes for cholesterol extraction assays were composed of [^14^C]PC/PE/PS/lactosyl-PE/[^3^H]cholesterol (59:20:10:10:1) and were prepared as described above.

Extraction assays were performed by incubating 20 μL of liposomes with 100 pmol protein and extraction buffer in a final assay volume of 80 μL for 20 minutes at 25°C. Liposomes were precipitated by incubation on ice with 10 μg *R*. *communis* agglutinin for 15 min and sedimented by centrifugation, and radioactivity in 50 μL of the supernatant containing extracted PI(4)P was measured by liquid scintillation counting. Liposomes were solubilized in 100 μl 1% (w/v) SDS in a bath sonicator and 50 μL of solubilized liposomes were measured by scintillation counting. Extraction is expressed as the percentage of radioactivity in the supernatant compared to the liposome pellet, and values are adjusted for background measured by conducting the experiment with no added protein.

### Gel filtration-high performance liquid chromatography (GF-HPLC)

Recombinant ORP4L-WT, ORP4L-S4A and ORP4L-S4D (25 μg) were resolved by GF-HPLC using a YARRA SEC-3000 silica column (7.8 mm x 300 mm, 3 μm particle size, 290 Å pore size) under isocratic conditions (10 mM HEPES, 150 mM KCl, pH 7.4) at a flow rate of 0.5 mL/min. Protein was detected by intrinsic fluorescence (λ_ex_ 285 nm, λ_em_ 335 nm) using a Waters 474 scanning fluorescence detector. To estimate protein mass based on retention time, the column was calibrated using carbonic anhydrase (29 kDa), BSA (66 kDa), β-amylase (200 kDa) and bovine thyroglobulin (669 kDa).

### Glutaraldehyde cross-linking

Purified wild-type ORP4L, ORP4L-S4A and ORP4L-S4D (1 μg) were incubated for 5 min at 37°C with glutaraldehyde (0–0.5%, w/v) in a final volume of 10 μL HEPES buffer (20 mM, pH 7.5). The crosslinking reaction was stopped by addition of 1 μL Tris (1 M, pH 8.5) and heating to 95°C in 2.5x SDS-PAGE reducing buffer. Proteins were resolved by SDS-6%PAGE and stained with GelCode Blue.

### Immunofluorescence microscopy

Cells cultured on glass coverslips were fixed with 4% (w/v) paraformaldehyde in PBS for 12 min at ambient temperature and then permeabilized for 12 min at 4°C in PBS with Triton X-100 (0.05%, w/v). Slides were blocked with PBS containing 1% (w/v) BSA and probed with antibodies diluted in the same buffer. Slides were rinsed with distilled water prior to mounting in Mowiol 40–88. Confocal immunofluorescence microscopy was performed on a Zeiss LSM 510 Meta laser scanning confocal microscope using Zen acquisition software (Zeiss).

### Immunoblotting and immunoprecipitation

Cell lysates were prepared in 10% w/v SDS, 10 mM β-mercaptoethanol, 20% v/v glycerol, 200 mM Tris-HCl, 0.05% w/v bromophenol blue (pH 6.8) and heated at 95°C for 5 min. Samples were resolved by SDS-8%PAGE, transferred to nitrocellulose membranes and blocked in a 4:1 mixture of Tris-buffered saline (TBS; 50 mM Tris, 150 mM NaCl, pH 7.4) and Odyssey blocking buffer (LI-COR Biosciences). Subsequent antibody incubations were performed in 4:1 TBS with 0.1% Tween-20 and Odyssey blocking buffer, and washes were performed with TBS. Membranes were visualized using the LI-COR Odyssey infrared imaging system and associated software.

To quantify phosphorylation of ORP4, HeLa cells transiently expressing wild-type or phospho-mutants of ORP4L or ORP4S were incubated in phosphate-free DMEM containing 2% FBS, 100 nM okadaic acid and [^32^P]PO_4_ (200 μCi) for 6 hours. Cells were harvested in lysis buffer (20mM Tris HCl [pH 7.4], 1mM EDTA, 1mM EGTA, 1mM Na pyrophosphate, 0.25% Triton X and 100nM okadaic acid) on ice for 15 min. Supernatants were prepared by centrifugation at 10,000x*g* for 10 min and incubated with a V5 monoclonal antibody for 16 h at 4°C. Immune complexes were isolated on protein A-Sepharose and resolved by SDS-8%PAGE. Gels were dried and exposed to film at -80°C for 6–12 h. The [^32^P]-labelled supernatants were also immunoblotted for ORP4L and ORP4S expression using a V5 monoclonal antibody. [^32^P]PO_4_ incorporation into wild-type and ORP4 phospho-mutants was determined by densitometry and expressed relative to total ORP4 protein expression (quantified by infrared imaging of immunoblots as described above).

### In vitro protein kinase assays

Protein kinase assays were performed using purified ORP4L and ORP4L-S4A (20 μg) as substrates in a final volume of 25 μL (Kinexus Bioinformatics Vancouver, BC). Samples were mixed with the kinases (10–50 nM each) and the reaction was initiated by the addition of 5 μl of [γ-^32^P ]ATP (0.8 μCi) and incubated at room temperature for 30 minutes. The assay was terminated by spotting 10 μL of the reaction mixture onto a multiscreen phosphocellulose P81 plate, which was washed 3 times for 15 minutes each in 1% phosphoric acid. Radioactivity on the P81 plate was measured in scintillation fluid using a Trilux scintillation counter.

## Results

### Phosphorylation of a proline-rich site in the ORP4 ORD

The ORD of human ORP4 contains 3 serine doublets interrupted by proline residues (S_762_SPSSPSS_769_) (Fig 1A). Alignment of this region of ORP4 from different species indicated that one of the SSP repeats is deleted in rodent homologues (Fig 1B). An alignment of human OSBP and ORPs revealed that the serine/proline-rich motif in ORP4 is absent in its closest paralogue OSBP as well as in ORP1L and ORP11, and the entire region containing the motif is absent in the remaining 8 ORPs (Fig 1C). Note that some *H*. *sapiens* ORP4L transcripts reported in the NCBI database begin at a methionine residue 40 amino acids prior to the predicted start site of the cloned cDNAs [15, 19]. Here, the amino acid numbering in sequence alignments is based on the reported cDNA sequence. Homology modeling of the ORP4 ORD based on the structure for Osh3p using SWISS-MODEL (Fig 1D) [20] predicts that the serine/proline-rich motif is in a 28-residue loop that interrupts β-sheet 16 and extends from the opposite side of the PI(4)P and sterol binding pocket of the ORP4 ORD.

Based on curated phospho-proteome data sets on public websites and published reports [22–24], 5 residues in the serine-rich motif are phosphorylated in human ORP4L, while 3 residues are phosphorylated in murine ORP4L. To confirm that this site is phosphorylated, human ORP4L-V5 was transiently expressed in HeLa cells, immunoprecipitated and resolved by SDS-PAGE, and subjected to tryptic digestion and liquid chromatography-tandem mass spectrometry (LC-MS/MS) analysis. Phosphorylation of serine 762, 763, 766 and 768 was detected, while phosphorylation of serine 765 was inconclusive (S1 Fig). The presence of this unique phosphorylation site in the ORP4 ORD prompted us to investigate its functional significance. Because multiple residues in the site are predicted to be phosphorylated, we used site-directed mutagenesis to convert serine 762, 763, 766 and 768 to aspartate to mimic a maximally phosphorylated state (ORP4L-S4D and ORP4S-S4D), and to alanine to mimic a constitutively dephosphorylated state (ORP4L-S4A and ORP4S-S4A) (Fig 1A).

**Fig 2:**
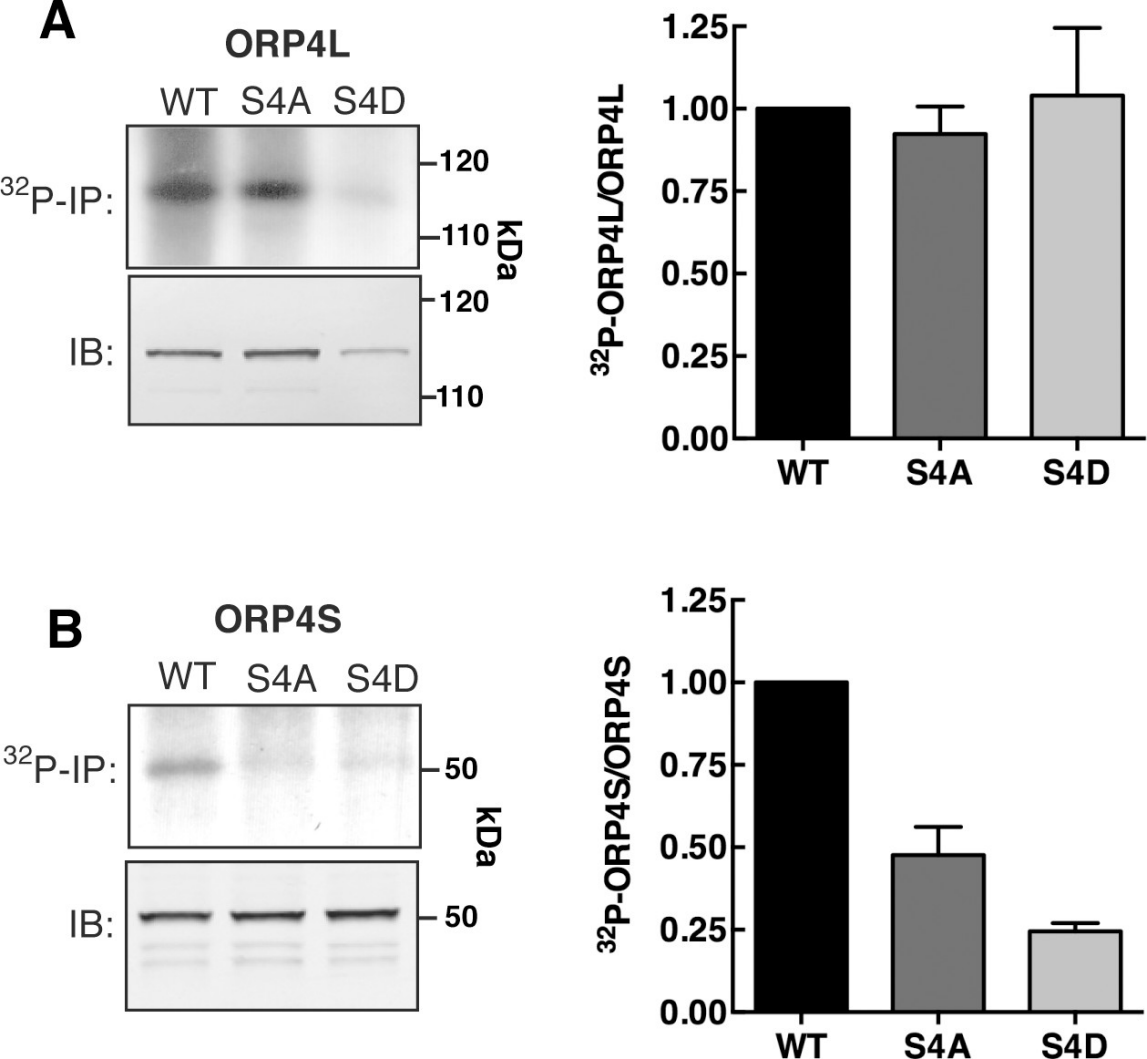
[^32^P]PO_4_ incorporation into ORP4L and ORP4S phospho-mutants. (**A**) Equivalent amounts of [^32^P]PO_4_-labeled extracts from Hela cells expressing ORP4L or ORP4S phospho-mutants were immunoprecipiated (^32^P-IP) with a V5 monoclonal antibody, resolved by SDS-PAGE and subjected to autoradiography. Total protein expression was determined by immunoblotting (IB) equivalent amounts of cell extract with a V5 monoclonal antibody. (**B**) Quantitation of [^32^P]PO_4_ incorporation is relative to total ORP4L or ORP4S protein. Results are the mean and SD of 3 experiments.

We assessed whether mutations in the serine/proline-rich motif affected global phosphorylation of ORP4L and ORP4S. HeLa cells transiently expressing wild-type and phospho-mutants were radiolabeled with [^32^P]PO_4_ and the proteins were subsequently immunoprecipitated, resolved by SDS-PAGE and identified by autoradiography (Fig 2). The incorporation of [^32^P]PO_4_ into ORP4L was not affected by S4A or S4D mutations when expressed relative to total ORP4 protein (Fig 2A). This result was not unexpected since ORP4L has 27 serine and threonine phosphorylation sites identified by high throughput proteomic analysis (https://www.phosphosite.org/) most which (16) are in the N-terminal region excluding the ORD. Since the serine/proline-rich motif in ORP4L could be minimally phosphorylated or obscured by phosphorylation in the N-terminal region, we examined the phosphorylation status of the ORP4S phospho-mutants. Wild-type ORP4S has fewer phosphorylation sites and incorporated approximately 20% less [^32^P]PO_4_ relative to ORP4L. Importantly, phosphorylation of the ORP4S-S4A and -S4D was reduced to 25–50% of wild-type levels (Fig 2B) indicating it is a major phosphorylation site in the ORD.

### In vitro phosphorylation of the ORP4 serine/proline-rich motif

**Fig 3:**
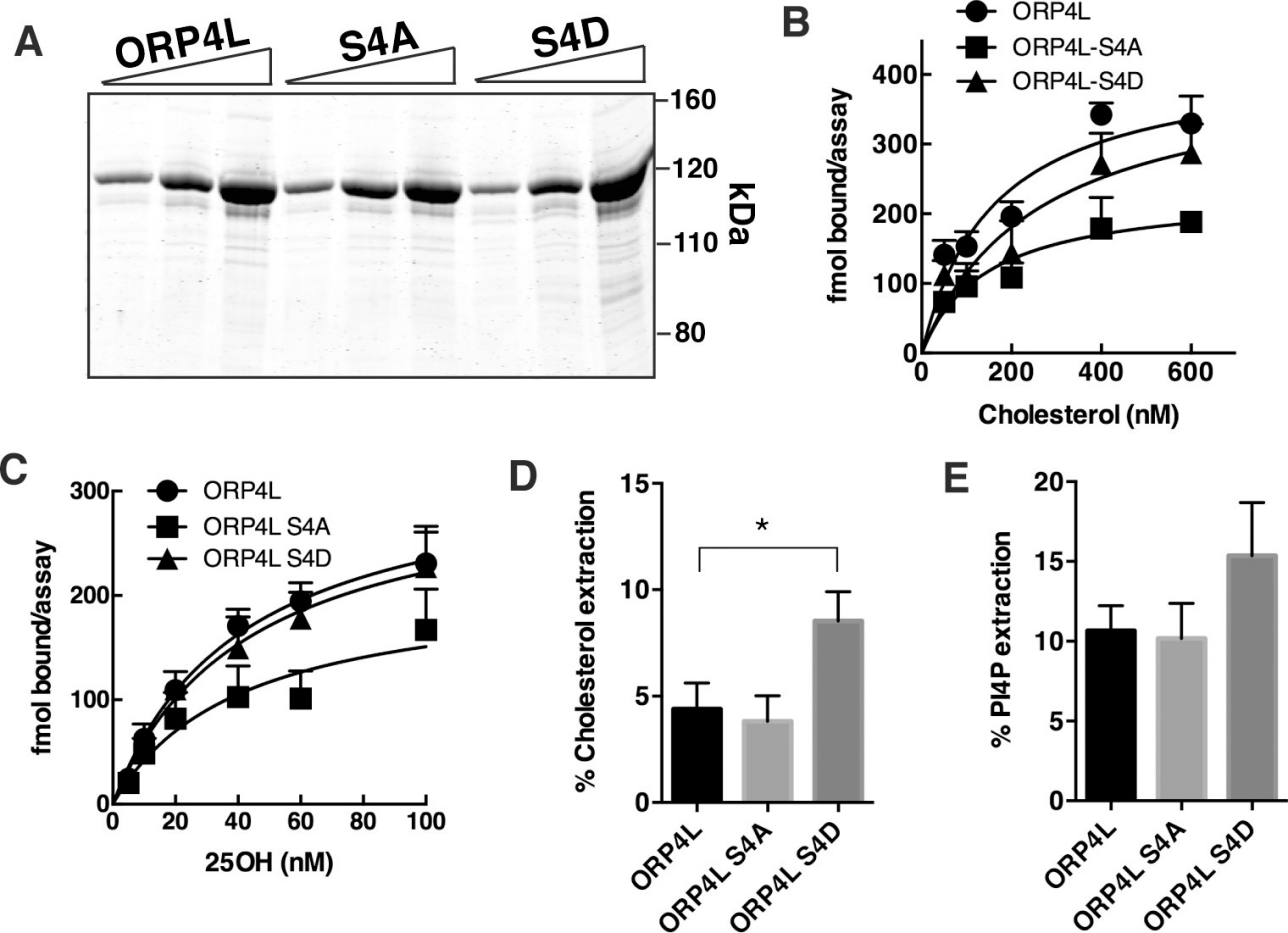
Phosphorylation of ORP4 affects cholesterol extraction from liposomes. (A) 6xHis-tagged wild-type ORP4L and the two phospho-site mutants were expressed in and purified from SF21 insect cells. Increasing amounts of protein (1, 2 and 5 μg) were resolved by SDS-8%PAGE and stained with GelCode Blue. (B-E) Recombinant ORP4L and phospho-site mutants were assayed for specific binding of [^3^H]cholesterol (B), specific binding of [^3^H]25OH (C), extraction of [^3^H]cholesterol from liposomes (D) and extraction of [^32^P]PI(4)P from liposomes (E) as described in Materials and Methods. Results in panels B-E are the mean and SEM of 3 experiments. *p<0.01.

The human ORP4 phosphorylation site is punctuated by two proline residues and was therefore predicted to be a substrate for proline-directed kinase(s). Using an *in silico* kinase prediction tool (PhosphoNET, Kinexus Bioinformatics), seven kinases were predicted to have the highest probability of phosphorylating serine 762, 763, 766 and/or 768; the proline-directed kinases glycogen synthase kinase (GSK) 3a and 3b, cJun N-terminal kinase 1 (JNK1), extracellular signal-regulated kinase 1 (ERK1) and cyclin-dependent kinase 1 (CDK1), as well as the non-proline-directed casein kinase 1 alpha 1 (CK1a1). These kinases were tested for their ability to phosphorylate ORP4L and ORP4L-S4A (purified from SF21 insect cells, Fig 3A) *in vitro* using [^32^P]ATP (Kinexus Bioinformatics Vancouver, BC). The amount of [^32^P]PO_4_ incorporation into ORP4L was expressed relative to ORP4L-S4A to identify those kinases with the highest activity and specificity for the phosphorylation site (Table 1). CK1a1, JNK1 and CDK1 had the highest site-specific activity for ORP4L, while CDK1, GSK3a, CK1a1 and GSK3b showed the highest specificity for the site when corrected for background activity with ORP4L-S4A. Because of the complexity of the serine/proline-rich site, we did not determine which serine(s) in ORP4L were phosphorylated by candidate kinases.

**Table 1:** *In vitro* phosphorylation of ORP4L and ORP4L-S4A by purified kinases.

Kinase	Substrate		Site-specific activity*	Specificity**
	ORP4L	ORP4L-S4A		
CDK1	17,218	7,310	9,908	2.4
GSK3a	7,745	4,096	3,649	1.9
CK1a1	48,238	30,044	18,194	1.6
GSK3b	11,948	9,093	2,855	1.3
JNK1	45,409	38,615	6,794	1.2
ERK1	10,423	9,080	1,343	1.2
JNK3	8,554	7,762	792	1.1

Results are from a representative experiment

* Site-specific activity = *ORP4L*_*activity*_*—ORP4L-S4A*_*activity*_.

** Specificity = *ORP4L*_*activity*_*/ORP4L-S4A*_activity_

### Effect of ORP4L phosphorylation on lipid binding

Recombinant His-tagged wild-type ORP4L and ORP4L-S4D and–S4A mutants were expressed using baculovirus in Sf21 cells and purified by metal-affinity chromatography (Fig 3A). The lipid binding activity of the recombinant phospho-mutants was then compared to wild-type ORP4L. The binding affinities of ORP4L (*K*_*d*_ 45±12 nM), ORP4L-S4A (*K*_*d*_ 62±28 nM) and ORP4L-S4D (*K*_*d*_ 60±18 nM) for [^3^H]25OH were not significantly different (Fig 3B). ORP4L (*K*_*d*_160±40 nM), ORP4L-S4A (*K*_*d*_ 255±90 nM) and ORP4L-S4D (*K*_*d*_ 144±43 nM) also had similar affinity for [^3^H]cholesterol (Fig 3C). The ability of ORP4L phospho-mutants to extract radiolabelled cholesterol and PI(4)P from membranes was measured using a liposome-based assay (Fig 3D and 3E). The extraction of [^3^H]cholesterol by ORP4L-S4D was increased 2-fold compared to wild-type and ORP4L-S4A, (Fig 3D). Extraction of [^32^P]PI(4)P from liposomes by ORP4L-S4D was increased slightly but did not reach significance (Fig 3D).

**Fig 4:**
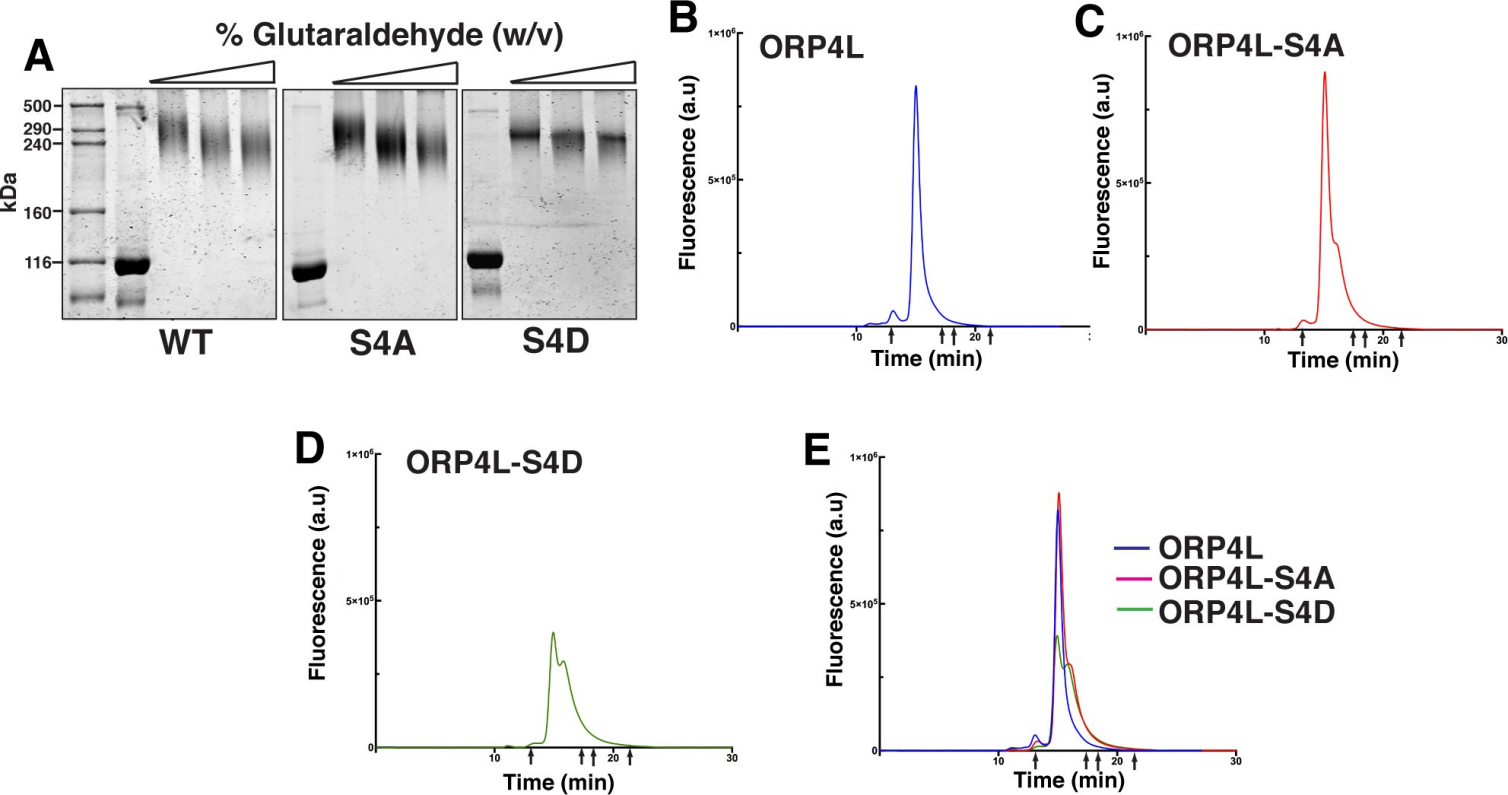
Effect of phosphorylation on the multimeric structure of ORP4L. (A) Recombinant ORP4L, ORP4L-S/D and ORP4L-S/A were incubated with increasing concentrations of glutaraldehyde (0–0.5%, w/v), separated by SDS-8%PAGE and stained with GelCode Blue. Similar results were observed in 4 other experiments. (B-E) ORP4L, ORP4L-S4D and ORP4L-S4A were resolved by GF-HPLC under isocratic conditions (arrows indicate the positions where standards eluted). The elution profiles are representative of 3 experiments.

Glutaraldehyde crosslinking and native gel filtration were used to determine the effect of phosphorylation on the oligomeric structure of ORP4L (Fig 4). Treatment of recombinant ORP4L and both phospho-mutants with increasing concentrations of glutaraldehyde shifted their mass on SDS-PAGE from 116 kDa to 240–290 kDa, which is just above that expected for a cross-linked dimer (Fig 4A). ORP4L was also resolved by gel filtration-HPLC (GF-HPLC) to determine the effect of phospho-site mutations on the native conformation (Fig 4B–4E). This technique revealed a major peak for ORP4L at ~450 kDa, indicating that the dimer has an extended rod-like shape compared to the globular protein standards used for column calibration or that native ORP4L adopts a tetrameric form not captured by cross-linking. ORP4L-S4A eluted as a major single peak with a small shoulder. ORP4L-S4D eluted as two poorly resolved species with a larger trailing shoulder (380–400 kDa), indicating that phosphorylation could alter the native conformation.

### Phosphorylation enhances the interaction of ORP4L with vimentin

**Fig 5:**
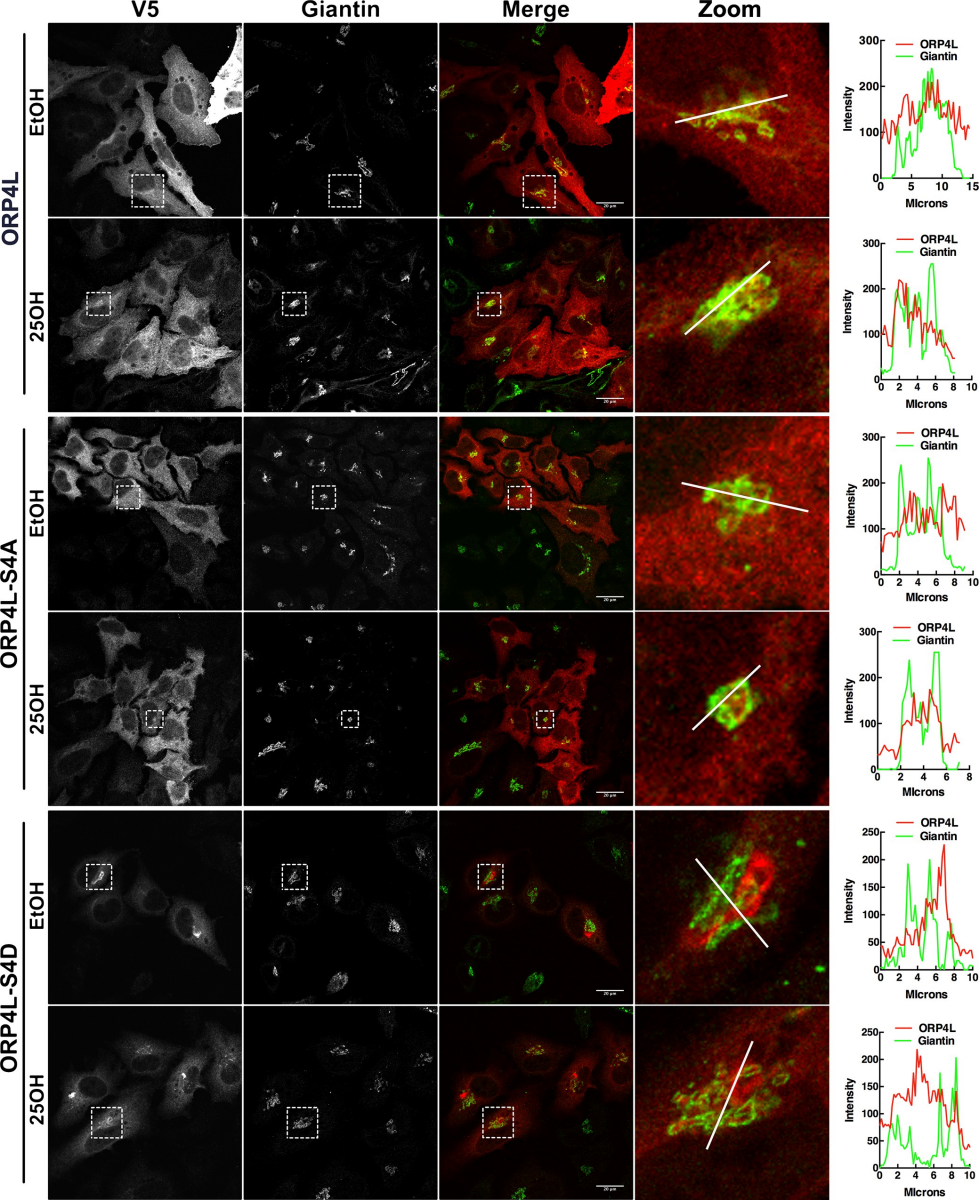
ORP4L-S4D localizes to the Golgi apparatus and perinuclear aggregates. HeLa cells transiently expressing ORP4L or phosphomimetics were treated with 25OH (6 μM) or control solvent for 2 h. Cells were fixed, permeabilized and immunostained with primary antibodies against V5 and giantin followed by secondary antibodies Alexa Fluor-594 and -488, respectively. Images are confocal sections (0.7μm). RGB line plots are shown for a selected region on the zoom image.

ORP4L has a complex distribution in cells, partitioning between the cytoplasm, ER, Golgi apparatus and PM [14, 15, 25]. In addition, N-terminal truncated ORP4S and ORP4M do not associate with the ER/Golgi/PM but instead interact with the vimentin intermediate filament network, causing it to bundle and aggregate in the perinuclear region [10]. To determine the effect of phosphorylation on intracellular localization, V5-tagged ORP4L-S4D or–S4A were transiently expressed in HeLa cells and imaged by immunofluorescence confocal microscopy (Fig 5). ORP4L and ORP4L-S4A were diffusely distributed in the cytoplasm but also displayed limited localization to the Golgi apparatus in response to 25OH treatment (see enlarged images). In contrast, ORP4L-S4D appeared in large perinuclear structures that did not overlap with the *cis/medial* Golgi marker giantin either in the presence or absence of 25OH. However, examination of enlarged images of 25OH-treated cells indicated that ORP4L-S4D was also present in smaller punctate structures that were closely associated with giantin.

**Fig 6:**
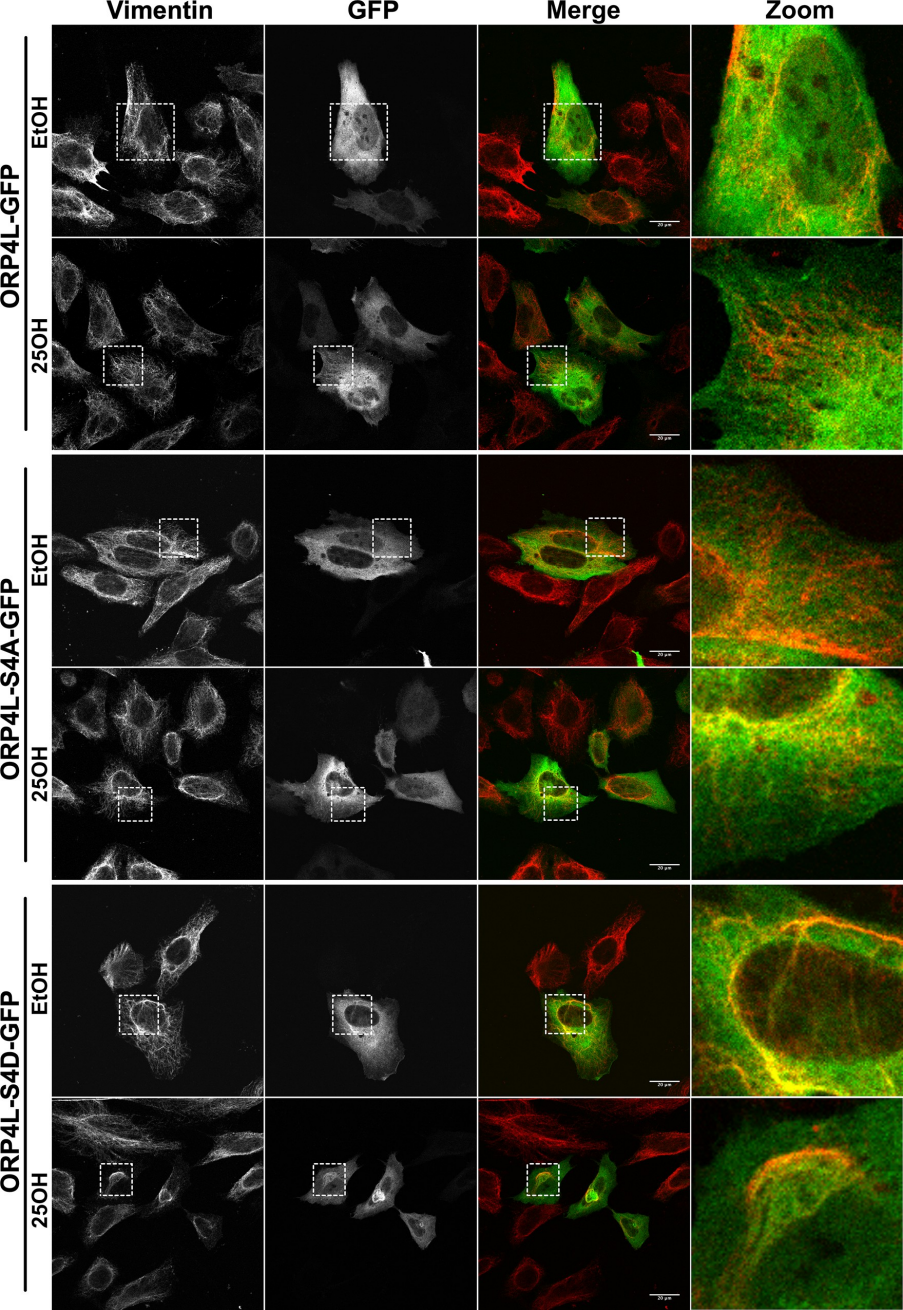
ORP4L-S4D co-localizes with an aggregated vimentin network. HeLa cells transiently transfected with GFP-tagged ORP4L or phosphomimetics were treated with 25OH (6 μM) or control solvent for 2 h. Cells were then fixed, permeabilized and probed with a primary antibody against vimentin followed by Alexa Fluor-594. Images are confocal sections (0.7μm).

**Fig 7:**
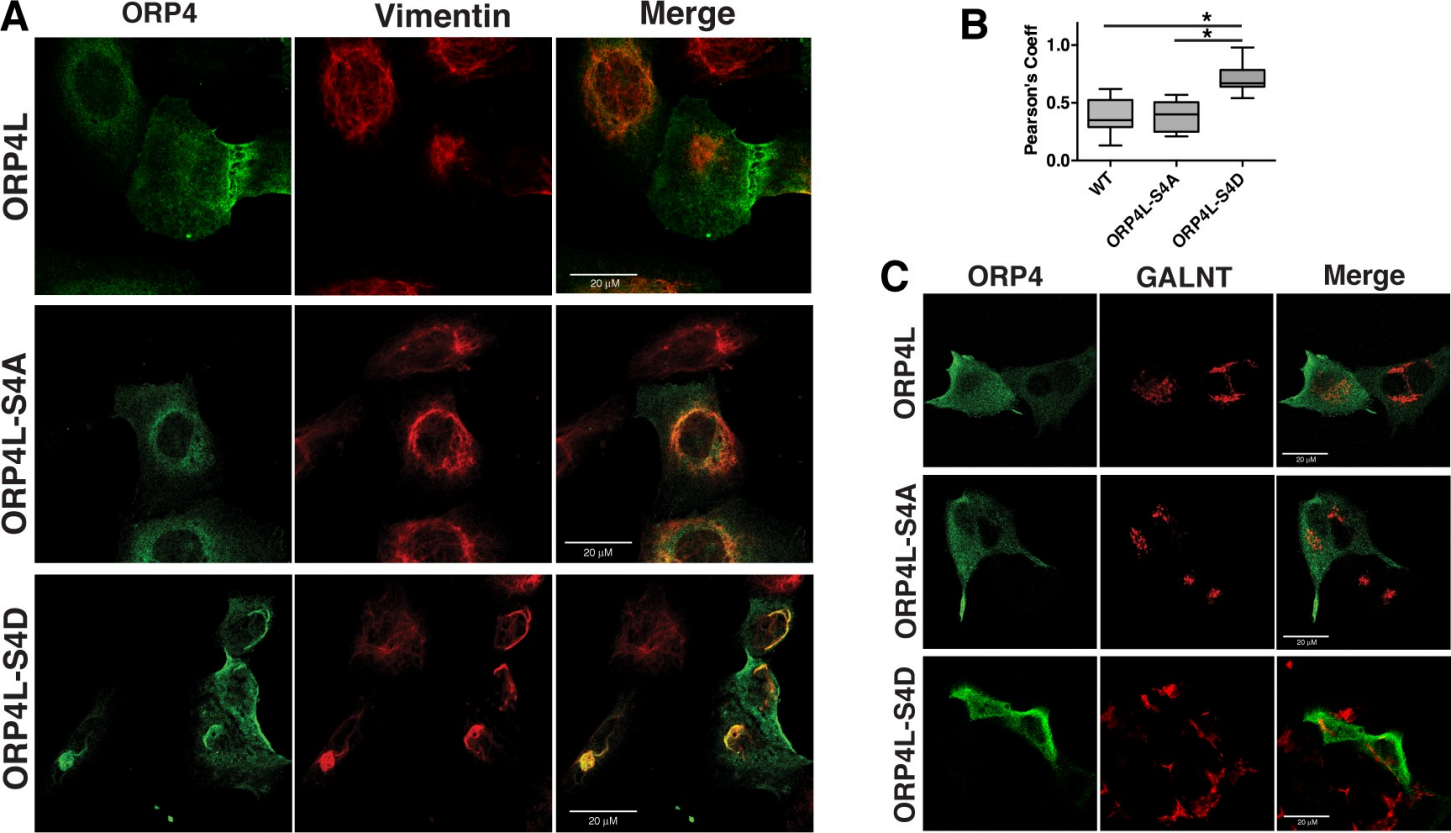
Intracellular aggregation of ORP4L-S4D is dependent on vimentin expression. (A) U2OS cells transiently expressing ORP4L and phospho-mutants were immunostained with an ORP4 polyclonal and vimentin monoclonal antibodies, followed by Alexa Fluor-488 and -594 secondary antibodies, respectively. (B) Pearson’s correlation coefficients for ORP4L and vimentin co-localization from panel A. Results are expressed as box and whisker plots (boxes indicate the interquartile range with bars at the median and whiskers at the 5^th^ and 95^th^ percentiles) for analysis of 20–30 cells (*p<0.001, one-way ANOVA). (C) HepG2 cells transiently expressing wild-type and ORP4L phospho-mutants were immunostained with an ORP4 polyclonal and GALNT monoclonal antibodies followed by Alex Fluor-488 and -594 secondary antibodies, respectively.

The structures formed by ORP4L-S4D resembled the aggregated vimentin filament network formed by ORP4S [15]. Thus, cells expressing green fluorescent protein (GFP)-tagged ORP4L and phospho-mutants were immunostained for endogenous vimentin. In HeLa cells, vimentin had a filamentous pattern that was unaffected by expression of wild-type or ORP4L-S4A, or treatment with 25OH (Fig 6). In cells expressing ORP4L-S4D, vimentin formed thick bundles or aggregates that co-localized with ORP4L-S4D. A similar result was observed in U2OS cells that also express abundant vimentin (Fig 7A). Expression of V5-tagged ORP4L and ORP4L-S4A in U2OS cells had no effect on the vimentin network while ORP4L-S4D was extensively co-localized with aggregated vimentin. V5-tagged ORP4L and the phospho-mutants were also expressed in HepG2 hematoma cells that lack vimentin expression (S2 Fig). In the absence of vimentin, ORP4L-S4D was diffusely localized in the HepG2 cytoplasm, indicating that ORP4L-S4D aggregation requires an existing vimentin network (Fig 7B).

**Fig 8:**
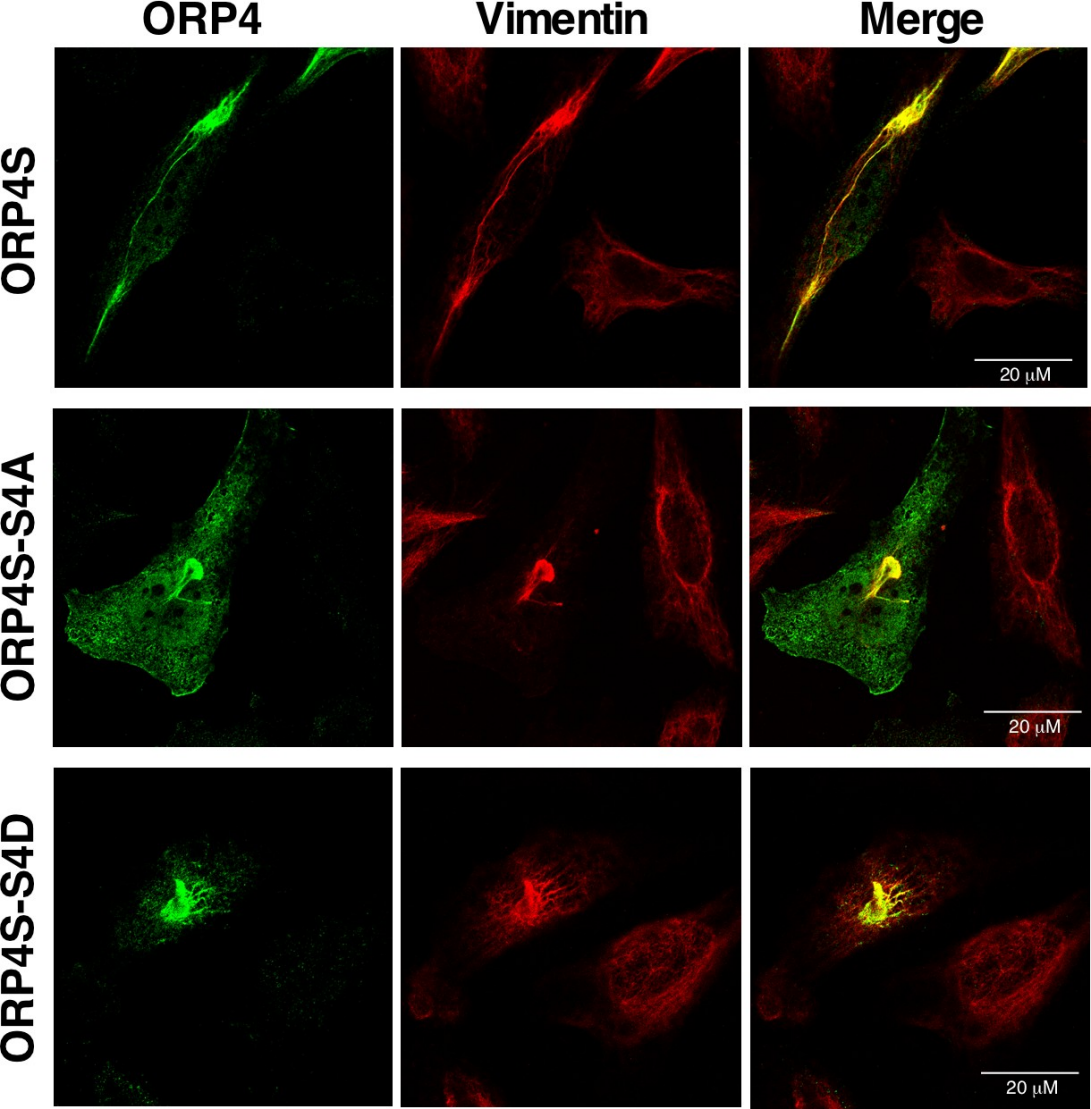
Phosphorylation of the serine/proline-rich motif in ORP4S does not affect vimentin aggregation. HeLa cells were transiently transfected with V5-tagged ORP4S, ORP4S-S4A or ORP4S-S4D. Cells were fixed, permeabilized and probed with primary antibodies against ORP4 and vimentin followed by secondary Alexa Fluor-594 and -488 antibodies, respectively.

Lastly, to determine whether the S4D mutation affected the ORP4/vimentin interface, the effect of the phospho-mutations on ORP4S localization was assessed by expressing the S4A and S4D mutants in HeLa cells. As expected, V5-tagged ORP4S was present in aggregates and filaments that co-localized with vimentin (Fig 8). The immunofluorescence localization of V5-tagged ORP4S-S4D and -S4A was indistinguishable from the wildtype protein, indicating the interaction is constitutive and unaffected by phosphorylation at the serine/proline-rich motif. Thus, phosphorylation of the serine/proline-rich site in ORP4L appears to induce a conformational change in ORP4L that mimics ORP4S, but the site is not directly involved in vimentin interaction.

## Discussion

To determine how phosphorylation of ORP4 regulates lipid binding activity and interaction with organelle membranes and vimentin, we searched for unique serine-rich sites in prior phospho-proteome screens that could be validated by LC-MS/MS and ^32^PO_4_ incorporation. A serine/proline-rich motif in the human ORP4 ORD, predicted to be in a solvent exposed loop opposite the lipid binding pocket, was identified and determined to be absent in OSBP and other ORPs, and partially deleted in rodent ORP4. *In silico* analysis and *in vitro* assays were used to identify CDK1, CK1a1, GSK3a and GSK3b as potential kinases with the highest specificity for the site. CDK1[26] and CK1a1 [27] are involved in cell cycle progression and as such their activity is temporally regulated. CK1a1, GSK3a and GSK3b are constitutively active, but phosphorylation by these kinases requires priming of the substrate by phosphorylation of an adjacent serine residue [28]. This suggests that the serine/proline-rich site in ORP4L isolated from insect cells could be partially phosphorylated or priming phosphorylation is not required. The phosphorylation of ORP4L by GSK3a and 3b could be relevant since deactivation of these kinases is required for progression of the epithelial-to-mesenchymal transition (EMT) and vimentin expression [29]. *In vitro* phosphorylation of ORP4L by JNK1 was notably high but only modestly reduced by the S4A mutation, suggesting phosphorylation by JNK1 at numerous serine-proline sites. This highlights the difficulty in identifying site-specific kinases using a substrate protein with numerous phosphorylation sites. Further investigation of signaling pathways that affect ORP4L phosphorylation and regulate vimentin interaction will provide insights into potential roles for ORP4 in cell proliferation [10], metastasis [30] and EMT.

Functional analysis of phosphomimetics of ORP4L indicated two major phenotypes associated with enhanced phosphorylation; increased cholesterol extraction from membranes and aggregation of vimentin intermediate filaments. Since the cellular localization and lipid binding activity of ORP4L-S4A was indistinguishable from wild-type ORP4L, it appears that the serine/proline-rich-motif is minimally phosphorylated under the conditions reported here. This conclusion is also supported by the lack of effect of S4A and S4D mutations on [^32^P]PO_4_ incorporation into ORP4L. On the other hand, ORP4L-S4D had increased cholesterol extraction activity from liposomes while its affinity for aqueous dispersions of 25OH and cholesterol was unaffected, implying that phosphorylation affects interaction with donor membranes but not the affinity of the lipid binding pocket for sterols. However, there was no obvious change in the association of ORP4L-S4D with the ER, Golgi or PM that might indicate enhanced lipid transfer activity at those sites. The most striking phenotype observed was enhanced association of ORP4L-S4D with aggregated vimentin. A fraction of ORP4L-S4D that was not in vimentin aggregates was localized to the Golgi apparatus in response to 25OH treatment. The enhanced interaction of ORP4-S4D with vimentin and cholesterol is similar to ORP4S, which constitutively associates with vimentin to induce perinuclear bundling of the intermediate filament network and has increased cholesterol extraction and transfer activity compared to ORP4L [10, 15]. The conclusion from those studies, that ORP4L is auto-inhibited by its N-terminal region with respect to both vimentin interaction and cholesterol extraction, can now be extended to include a phospho-switch in the ORP4L ORD that may regulate auto-inhibition. Indeed, a similar mechanism controls ceramide transport protein (CERT) interaction with membranes [31]. An intramolecular interaction between the PH domain and a serine-rich phosphorylation site in the linker region adjacent to the StAR-related lipid transfer domain is relieved by dephosphorylation, freeing the CERT PH domain to bind PI(4)P in the Golgi apparatus. In the case of ORP4L, phosphorylation does not appear to enhance PH domain interaction with membranes since we did not observe increased Golgi or PM localization of ORP4L-S4D. Instead, phosphorylation of ORP4L may release an intramolecular interaction between the ORD and the N-terminus of dimeric ORP4L, exposing a vimentin binding site in the ORD. Because both ORP4S phospho-mutants associated with vimentin, phosphorylation of the serine/proline-rich motif is not involved directly in the interaction.

ORP4L has not been directly implicated in lipid transfer at membrane contact sites but has protein partners that facilitate its recruitment and activity at the PM, Golgi apparatus and intermediate filaments [10, 12, 14]. This study shows that these interactions can be selectively regulated by phosphorylation, potentially shifting the cellular distribution and activity of ORP4L.

## Supporting information

S1 Fig LC-MS/MS analysis of the doubly-charged peptide containing the ORP4 phospho-site. ORP4L was subjected to trypsin digestion and LC-MS/MS analysis as described in the Materials and Methods, and peptide corresponding to amino acids 760–772 was identified using the Sequest HT DB search engine. A first fragmentation shows that the mass of the **(A)** unmodified peptide 760–772 differs from the mass of the (**B)** phosphorylated peptide 760–772 by one phosphate molecule (80 Da). Sequence-related information from a second fragmentation step confirmed individual phosphorylation of serine 762, 763, 766 and 768 (data not shown).
(TIF)

S2 Fig Vimentin expression in HeLa, U2OS and HepG2 cells. Whole cell lysates were immunoblotted with a vimentin monoclonal and OSBP polyclonal antibodies, followed by IRDye 680LT- and 800CW-conjugated secondary antibodies.
(TIF)
