# Supplementary figures and images for: Phosphorylation of a serine/proline-rich motif in oxysterol binding protein-related protein 4L (ORP4L) regulates cholesterol and vimentin binding
Source: PLoS One. 2019 Mar 29;14(3):e0214768. doi: 10.1371/journal.pone.0214768 (PMC6440634; doi:10.1371/journal.pone.0214768)

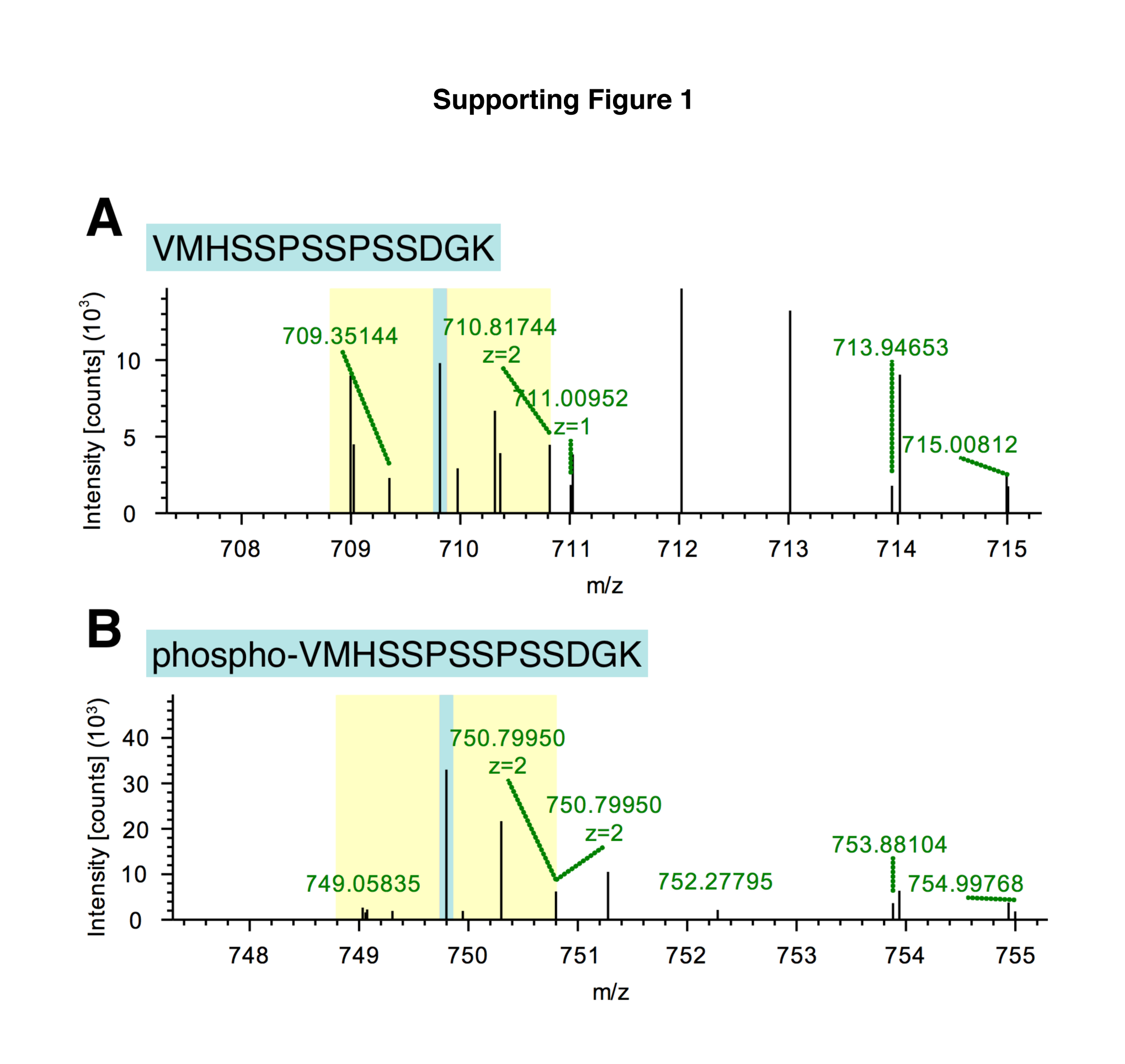

Supplement: S1 Fig — ORP4L was subjected to trypsin digestion and LC-MS/MS analysis as described in the Materials and Methods, and peptide corresponding to amino acids 760–772 was identified using the Sequest HT DB search engine. A first fragmentation shows that the mass of the (A) unmodified peptide 760–772 differs from the mass of the (B) phosphorylated peptide 760–772 by one phosphate molecule (80 Da). Sequence-related information from a second fragmentation step confirmed individual phosphorylation of serine 762, 763, 766 and 768 (data not shown). (TIF) [file pone.0214768.s001.tif]

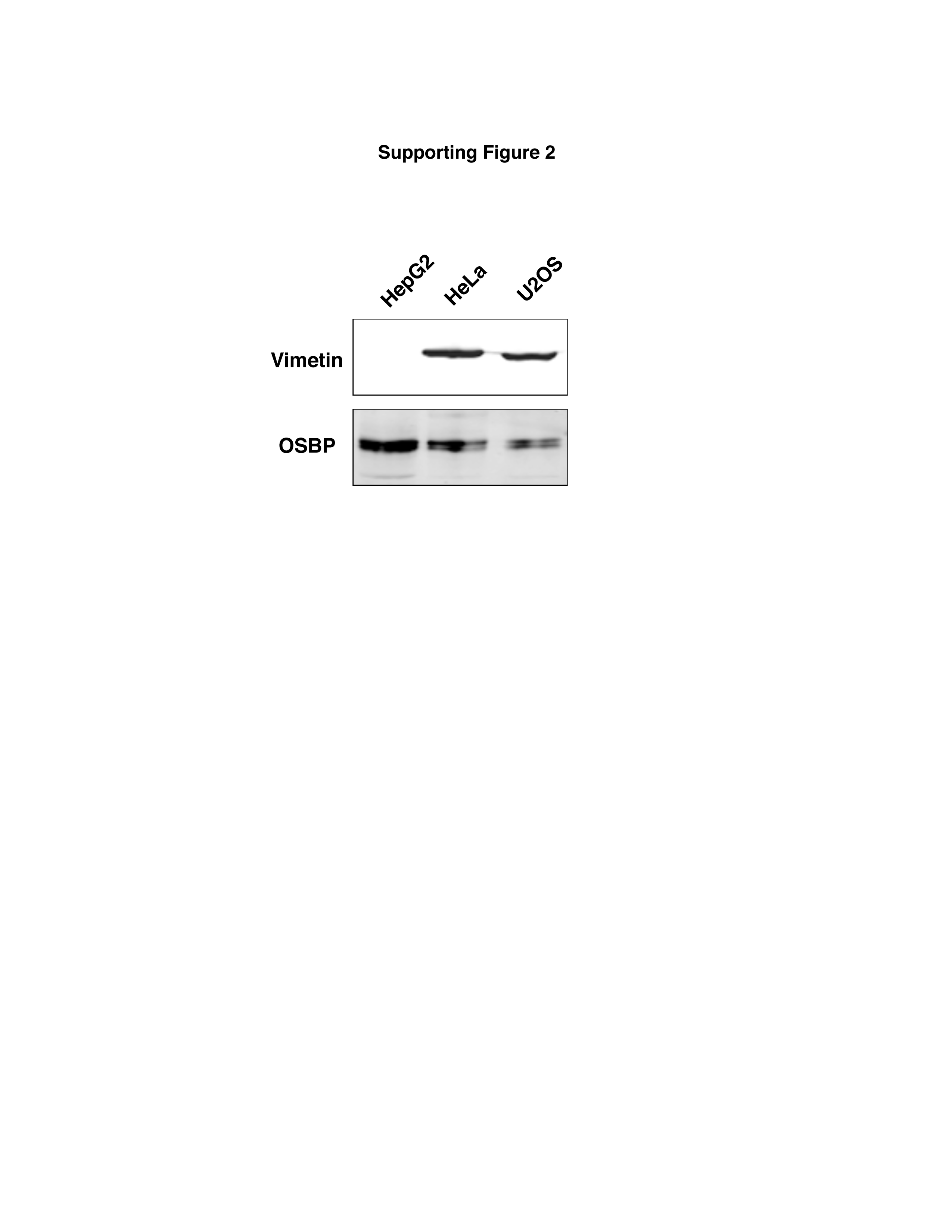

Supplement: S2 Fig — Whole cell lysates were immunoblotted with a vimentin monoclonal and OSBP polyclonal antibodies, followed by IRDye 680LT- and 800CW-conjugated secondary antibodies. (TIF) [file pone.0214768.s002.tif]
